# Tropical Heart

**Published:** 1898-09-03

**Authors:** 


### TROPICAL HEART.

The *Journal of Tropical Medicine*, the first number of which has been issued this week, has at once stepped out of the rut by publishing a communication from Surgeon-Colonel K. Macleod on "Tropical Heart." So much attention has of late been devoted to malaria and other parasitic and zymotic tropical diseases, that it is well we should be reminded how much other morbid conditions are apt to be modified and even caused by the climatic and other surroundings of life in the tropics. We are all accustomed to what has so long been described as soldiers' heart. What, however, is important to bear in mind is the still further element of strain, in addition to what is involved by military training, which is introduced by residence and work in the tropics. To such an extreme does this go that the "Madras heart" has become a well-known affection in the sout hern presidency, and is by no means confined to the latitude of uninterrupted heat. It is found in acute and chronic forms through India. The lesion is precisely the same as the "disordered action," for which so many soldiers are sent home. The moral drawn by the author of the paper is, that in examining persons, especially adolescents, for tropical employment, very particular care should be exercised in taking stock of the condition of the heart and circulation. None but sound hearts are fit to encounter and withstand the strain which the new and strange environment imposes on both the central organ and vessels. The *Journal of Tropical Medicine* has done well to give prominence to this article, for, as it truly says, the wreck of sound hearts which takes place in the army, especially in the tropics, is a serious matter, which demands not only care in selection of recruits, but inculcates also continued solicitude in matters of training and drill.

